# Assaying Visual Memory in the Desert Locust

**DOI:** 10.3390/insects6020409

**Published:** 2015-04-20

**Authors:** Senne Dillen, Ziwei Chen, Jozef Vanden Broeck

**Affiliations:** Molecular Developmental Physiology and Signal Transduction, Department of Biology, KU Leuven, Leuven 3000, Belgium; E-Mails: senne.dillen@bio.kuleuven.be (S.D.); ziwei.chen@student.kuleuven.be (Z.C.)

**Keywords:** locust, visual memory, learning, sNPF

## Abstract

The involvement of associative learning cues has been demonstrated in several stages of feeding and food selection. Short neuropeptide F (sNPF), an insect neuropeptide whose effects on feeding behavior have previously been well established, may be one of the factors bridging feeding and learning behavior. Recently, it was shown in *Drosophila melanogaster* that the targeted reduction of *Drome*-sNPF transcript levels significantly reduced sugar-rewarded olfactory memory. While *Drosophila* mainly relies on olfactory perception in its food searching behavior, locust foraging behavior is likely to be more visually orientated. Furthermore, a feeding-dependent regulation of *Schgr*-sNPF transcript levels has previously been observed in the optic lobes of the locust brain, suggesting a possible involvement in visual perception of food and visual associative memory in this insect species. In this study, we describe the development of a robust and reproducible assay allowing visual associative memory to be studied in the desert locust, *Schistocerca gregaria*. Furthermore, we performed an exploratory series of experiments, studying the role of *Schgr*-sNPF in this complex process.

## 1. Introduction

Although the main role of short neuropeptide F (sNPF) seems to lie in the regulation of feeding behavior [1,2,3], it has been implicated in many other physiological processes, ranging from developmental processes such as molting [4] and the regulation of reproduction [5], to sleep homeostasis and the maintenance of circadian rhythms [6,7]. In *Drosophila melanogaster*, however, sNPF was also shown to be involved in the regulation of olfactory memory and learning behavior [8,9].

One of the major structures in the insect brain known to be involved in higher olfaction, learning and memory are the paired mushroom bodies [10]. In *Drosophila melanogaster*, there are about 2500 intrinsic mushroom body cells, the Kenyon cells, and a large number of extrinsic cells, connecting the various sections of the mushroom bodies. The dendrites of these Kenyon cells form a globular microglomerular complex called the calyx, which receives the main olfactory input from the antennal lobes by means of olfactory projection neurons [9,11]. In 2008, Nässel and coworkers used a combination of *in situ* hybridization and immunocytochemistry to demonstrate that the vast majority of these Kenyon cells express *Drome*-sNPF [12]. More recently, it was shown by Knapek *et al.* that the targeted reduction of *Drome*-sNPF transcript levels in these Kenyon cells significantly reduced sugar-rewarded olfactory memory. Additionally, knockdown of the *Drome*-sNPF receptor transcript outside of the mushroom bodies caused a reduction in appetitive memory, indicating that sNPF likely acts as a neurotransmitter, carrying a signal from the intrinsic to the extrinsic cells [9]. It has previously been shown that food-associated memory is very closely linked to the motivational state of the animal. Studies performed in *Drosophila melanogaster* showed that when the *Drome*-NPF transcript is knocked down in normally fed flies, mimicking the starved state, memory performance is significantly improved [13]. Furthermore, it has been shown that *Drome*-sNPF, expressed in olfactory receptor neurons in the antennal lobes of the *Drosophila* brain, can take up a neuromodulatory function, modulating the fly’s perceptiveness of olfactory cues depending on its nutritional state. This increase in olfactory perceptiveness is very likely to further increase the efficiency of olfactory learning in *Drosophila* flies [14].

While *Drosophila* may mostly depend on olfactory cues when engaging in food-searching behavior, it is unsure whether this is also the case in *Schistocerca gregaria*. In the swarming gregarious phase, which mostly occurs in environmental conditions in which the availability of food is low, these animals can engage in long-distance flights when foraging for food. In such circumstances, visual cues might gain importance in guiding the animals towards a possible food source. Furthermore, while *Drome*-sNPF transcript levels are regulated in the antennal lobe are regulated according to the nutritional state of the animal, the abundance of the *Schgr*-sNPF mRNA in the optic lobes of the locust brain is subjected to a similar nutrient-dependent regulation [3]. For these reasons, we designed an assay allowing visual memory to be assayed in *Schistocerca gregaria*, and studied whether in this locust species, it is visual rather than olfactory memory that is affected by sNPF signaling.

## 2. Experimental Section

### 2.1. Animal Rearing Conditions

The desert locusts used in this study were all gregarious animals in the fifth larval stage. They were reared under crowded conditions at a constant temperature (30 ± 2 °C) and photoperiod (14 h). The locusts were kept at high density (>200 locusts per cage) and fed daily with cabbage and dry oat flakes. Experimental and control groups contained equal numbers of male and female animals.

### 2.2. Experimental Arenas

**Figure 1:**
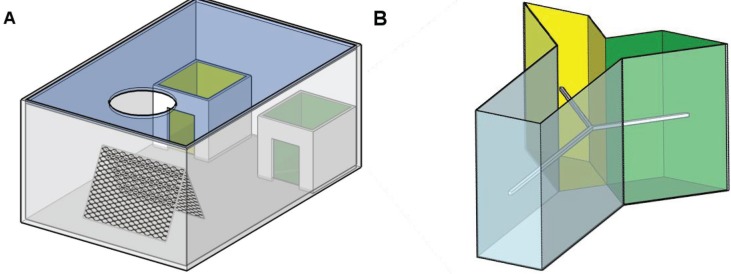
(**a**) The training arena is a rectangular cage with two smaller cages at the far end. The insides of these smaller cages are lined with either yellow or green laminated paper; (**b**) The Y-maze consists of a branch and two smaller side branches, covered with yellow or green laminated paper. Throughout the Y-maze, a wooden rod is suspended on which the animals are allowed to move.

To assay locust learning behavior, two separate arenas were used. One of these was based on the design used by Bernays and Wrubel and will be referred to as the training arena [15]. This training arena is a rectangular cage measuring 20 × 30 × 45 cm. The walls were constructed of white PVC, while the top surface was made out of acrylic glass. The bottom surface consisted of a sheet of laminated paper. Inside this arena, two open-topped smaller cages of 10 × 11 × 10 cm were placed, the inside of which was covered in either green or yellow laminated paper. On the side opposing the two smaller boxes, a triangular structure consisting of wire mesh was placed, which is referred to as the roost. Above the roost, a high intensity lamp (60 W tungsten bulb) was suspended, providing a basking spot for the locusts. Between the roost and the smaller cages, a removable partition is present (Figure 1A). The second arena was based on the design used by Niven and coworkers [16]. This is a Y-maze constructed of white PVC, with a top surface of acrylic glass. This Y-maze consists of one main branch measuring 10 × 22 × 24 cm and two side branches measuring 10 × 22 × 15 cm. The insides of these two side branches were covered with either green or yellow laminated paper. Throughout the length of the Y-maze, a wooden Y-shaped rod was suspended on which the animals were allowed to move (Figure 1B).

### 2.3. Teaching Setup

Two series of training experiments were performed. In the first, both the training period and the assessment of the efficiency of training were done using the training arena. In the second series of experiments, training was performed in the training arena while the efficiency of training was assessed in the Y-maze.

#### 2.3.1. Training Arena as Assessment Arena

Naive locusts in the fifth larval stage were starved overnight and placed on the roost of the training arena. The animals were then left undisturbed for 30 min, allowing them to adjust to their new environment. When these 30 min had past, the PVC sheet separating the roost from the rest of the training arena was removed and the animals were allowed to move freely throughout the training arena. As the animals were starved overnight, they would periodically leave the roost to wander the arena in search of food. During these bouts of food-searching behavior, they would visit both green and yellow cages. For a period of five hours, it was monitored which cages were visited by the animals. Furthermore, it was monitored how long each animal spent in each of these cages. Based on these data, a preference index was calculated for each individual animal (equations 1 and 2), where a preference index of 1 indicates absolute preference for the yellow cage, while a preference index of 0 indicates absolute preference for the green cage.

(1) $$Pref_{time}=\;\frac{time\;spent\;in\;yellow\;cage}{total\;time\;spent\;in\;yellow\;or\;green\;cage}$$

(2) $$Pref_{visits}=\;\frac{number\;of\;visits\;to\;yellow\;cage}{total\;number\;of\;visits\;to\;yellow\;or\;green\;cage}$$

As it was consistently shown that the average preference index was greater than 0.5, indicating that naive animals preferred the yellow over the green cage, they were subsequently trained to visit the green cage. This was done by placing several pieces of cabbage in the green cage and allowing the animals to freely move throughout the training arena overnight. To ensure the perception of visual cues, the light above the roost was left on throughout this training period. When the animals would visit the green cage, they would encounter the food within, which would act as a positive stimulus. After this period of overnight training, all animals were again placed on the roost and the food was removed from the arena. To eliminate any possible odor traces, the colored lining of the cages was replaced, as was the laminated paper covering the bottom of the arena. To exclude any effects of positional learning, the position of the yellow and green cages were switched. After the animals had been left on the roost for one hour, the PVC sheet separating them from the rest of the arena was once again removed and their movement was monitored for another period of five hours.

Based on the preference indexes calculated before and after training, the training efficiency was determined for each individual locust, as shown by the equation (3) below.

(3) $$Training\;efficiency=\;\frac{Preference\;index\;\left(untrained\right)-Preference\;index\;\left(trained\right)}{Preference\;index\;\left(untrained\right)}$$

#### 2.3.2. Y-Maze as Assessment Arena

In this series of experiments, preference indexes were determined by using a Y-maze rather than the training arena. Naive animals were placed on the wooden rod suspended in the Y-maze and, when moving forward, would make a choice between entering either the green or yellow branch. Based on these visits, the preference index was calculated using the equation (4) below.

(4) $$Pref_{\;visits}=\;\frac{number\;of\;visits\;to\;the\;yellow\;branch}{total\;number\;of\;brach\;visits}$$

After five measurements had been made for each animal, training was performed in the training arena as described in the previous section. After training, each animal was once more placed in the Y-maze and tested four times. Similarly as in the first series of experiments, the efficiency of training was determined by comparing preference indexes before and after training.

### 2.4. sNPF Precursor Transcript Knockdown

Five days prior to the onset of the experiments, male and female animals in the fifth larval state were dorsally injected with 2 µL of a 100 ng/µL solution of dsRNA. Animals in the experimental group were injected with dsRNA directed against the *Schgr*-sNPF precursor transcript while control animals were injected with an equal amount of dsRNA directed against GFP. The generation of these dsRNA fragments is discussed in detail in previously published work [3].

Five days after injection, the experimental and control animals were simultaneously placed in the training arena and their training efficiency was determined as described in Section 2.3.1.

## 3. Results

### 3.1. Assaying Locust Visual Memory

**Figure 2:**
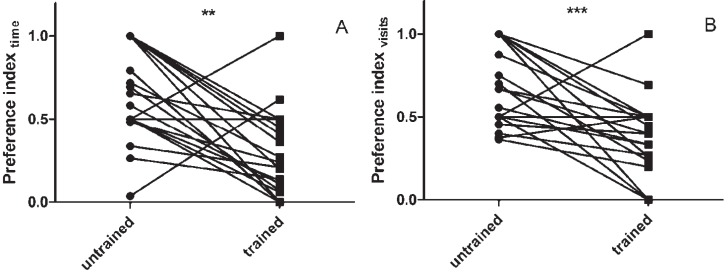
The effect of training on color-dependent foraging behavior, as assayed in the training arena. Untrained animals generally preferred the yellow cage (Pref > 0.5). Overnight training significantly reduced both the time spent in the yellow cage (**A**) as the relative number of visits to this cage (**B**). Data represent paired measurements before and after training (*n* = 25). Results were analysed using the Wilcoxon signed rank test. ** *p*<0.01, *** *p*<0.001.

Using the training setup described in Section 2.3.1, in which the training arena was used for both training and the assessment of learning behavior, we were able to induce a clear change in locust food-searching behavior (Figure 2).

**Figure 3:**
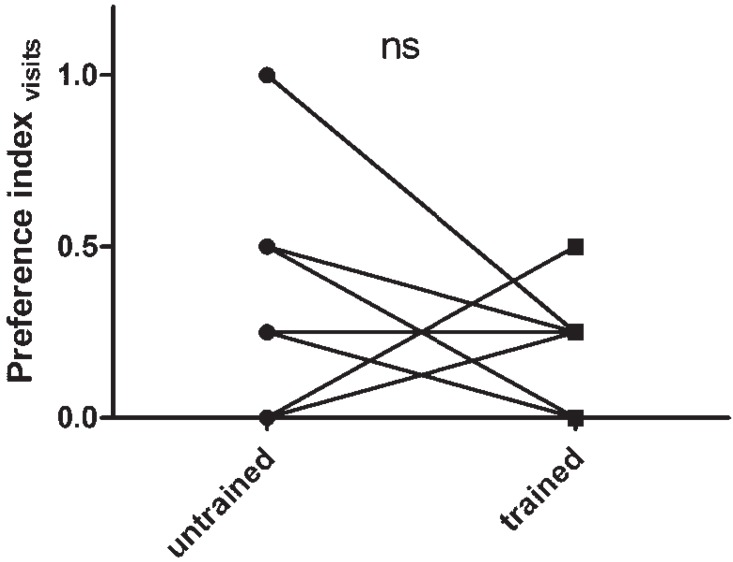
The effect of training on color-dependent foraging behavior, as assayed in a Y-maze. When assessments before and after learning are made in a Y-maze, overnight learning cannot be shown to effect branch-choice. Data represent paired measurements before and after training (*n* = 19, the seemingly low sample size is due to overlapping data points). Results were analyzed using the Wilcoxon signed rank test. ns: no significant difference.

When the pre- and post-training assessments were performed in the Y-maze, however, no clear shift in color-dependent foraging behavior was observed (Figure 3).

### 3.2. The Effect of sNPF on Visual Memory

**Figure 4:**
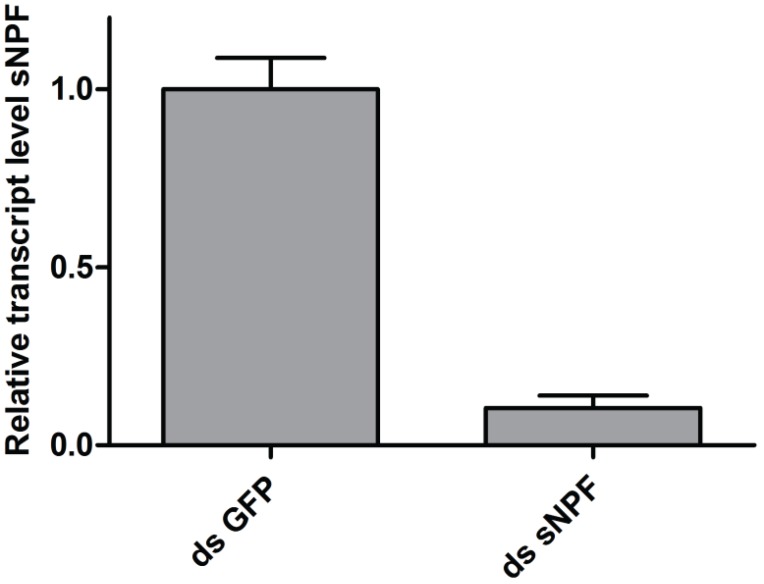
Relative *Schgr*-sNPF precursor transcript levels in the optic lobes of the brain upon *Schgr*-sNPF knockdown. Locusts were injected with 200 ng dsRNA corresponding to either GFP or sNPF. Injection of sNPF dsRNA resulted in an 89% reduction in transcript levels five days after injection. Data represent mean values ± SEM (*n* = 6).

As these previous experiments showed that locust learning behavior can readily be assayed using the setup outlined in Section 2.3.1, using the training arena both for training as for pre- and post-training assessment, this setup was used to study whether sNPF influences visual learning in desert locusts. Locusts were injected with 200 ng of dsRNA corresponding to the *Schgr*-sNPF precursor, which reduced the precursor transcript levels in the optic lobes by 89% (Figure 4). Training efficiencies were calculated for experimental animals, in which the *Schgr*-sNPF precursor transcript had been knocked down, and control animals, injected with dsRNA directed against GFP. The result of these experiments is shown in Figure 4.

Although the mean values for training efficiency of the *Schgr*-sNPF knockdown group are lower for both methods of calculation, no significant differences are observed between the control and experimental groups. These data clearly show that using the current setup, knockdown of the *Schgr*-sNPF precursor transcript could not be shown to significantly affect the efficiency of color-dependent visual learning in locusts in the fifth larval state, neither when it comes to efficiency measured by the number of visits, nor when it comes to the efficiency measured by the total time spent in each cage.

## 4. Discussion

It has previously been shown that sNPF affects olfactory perceptiveness and olfactory memory in *Drosophila* *melanogaster*, enabling the animal to optimally exploit the available food sources [9,14]. In this study, we investigated whether or not similar processes are also present in the desert locust, albeit based on visual rather than olfactory perception.

To assay these putative shifts in learning efficiency, an assay had to be developed which allows the visual memory and learning behavior of *Schistocerca gregaria* to be studied in a straightforward and reproducible manner. The selected training method was based on the one used by Bernays and Wrubel, optimized for the color-dependent training of *Melanoplus sanguinipes* larvae [15]. For this series of experiments, animals in the fifth larval stage were selected, as fifth stage larvae of *Melanoplus sanguinipes* were shown to possess highly efficient color-dependent learning responses, while positional-learning responses, which might interfere with the experimental setup, were shown to decline after the fourth larval stage [15].

It is interesting to note that when naive locusts or grasshoppers engage in foraging behavior and are given a choice between entering a green or yellow container, all of the relevant literature indicates that the majority of animals display a preference for yellow [15,17,18]. Based on this knowledge, which was corroborated by our own observations (Figure 2), a positive stimulus—in the form of food—was placed in the green cage, and the animals were trained to preferably visit this green cage when engaging in food-searching behavior. 

To assess the efficiency of training, two separate assessment arenas were used. One of these consisted of a Y-maze, previously used to assess olfactory learning in *Schistocerca gregaria* [16,19]. In this setup, animals are forced to make a choice between one of two different colored branches. While this enables locusts to be studied relatively quickly and individually, excluding possible group-effects, the forced nature of the choice might make this setup unsuitable for assaying changes in spontaneous food-searching behavior. Furthermore, the animals were tested multiple times in unrewarded trials, likely leading to a further reduction of the link between color-preference and foraging behavior. It is likely that because of these reasons, overnight training could not be shown to have an effect in this experimental setup (Figure 3).

A more suitable, although highly labor intensive, approach was the one proposed by Bernays and Wrubel, in which the same arena was used for both training and the assessment of training efficiency [15]. When pre- and post-training behavior was observed using this setup, a clear and highly significant shift in color-preference can be observed towards the color associated with the positive stimulus (Figure 2).

**Figure 5:**
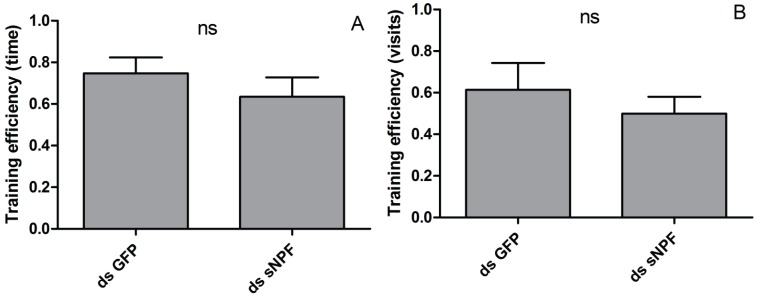
RNAi mediated knockdown of the mRNA encoding the Schgr-sNPF precursor does not significantly influence the efficiency of visual learning. Training efficiencies were calculated according to the time spent in each box (**A**) or the total number of visits to each box (**B**) Data represent average ± SEM (*n* = 6). Results were analyzed using the Mann-Whitney U test. ns: no significant difference.

It has previously been described that the efficiency of learning and memory in an animal is closely tied to its motivational state [13,20]. In light of this, it does not surprise that when the transcript encoding the sNPF precursor was knocked down in *Drosophila melanogaster*—mimicking a well-fed state, in which little priority is given to the efficient localization of food-sources—a reduction of memory performance was observed [9]. Analogous to this, we hypothesized that knocking down the sNPF precursor transcript in *Schistocerca gregaria*—mimicking the starved state, in which animals are highly motivated to engage in food-searching behavior—might cause an increase in the efficiency of learning and memory. When this hypothesis was put to the test, however, no experimental data could be obtained to indicate that *Schgr*-sNPF might negatively influence visual memory of efficiency or visual learning (Figure 5). When we look at the number of animals leaving the roost, however, a trend does seem to emerge. During the pre-learning observation period, 25% of the animals in the control group did not leave the roost, compared to 46% of the animals in the experimental group. In the post-learning observation period, these values shifted to respectively 15% and 27%. Although this very limited dataset is insufficient to draw any final conclusions, it does seem that knocking down the *Schgr*-sNPF transcript reduces the animals’ tendency to engage in food-searching behavior. This is counterintuitive because *Schgr*-sNPF acts as a satiety signal and inhibits feeding, as discussed in Dillen *et al.* [2,3]. A possible explanation might be found in experiments performed in *Drosophila melanogaster*, where it was shown that *Drome*-sNPF is expressed in the lateral clock neurons of the *Drosophila* brain. These lateral neurons act as the principal oscillators regulating the bouts of activity in the morning and evening [6,21]. As locusts tend to feed in the morning and the evening, the decrease in foraging behavior might be due to the disruption of sNPF signaling in these lateral clock neurons. Another possibility is that locusts in which the sNPF transcript has been knocked down will display an upregulation of daytime sleep, as was previously demonstrated in *Drosophila melanogaster* [7]. If these animals sleep more, it stands to reason that the likelihood of engaging in foraging behavior will decrease.

In conclusion, although we were not able to demonstrate an effect of *Schgr*-sNPF on visual memory, inhibitory or otherwise, we have designed a robust method for assaying the effect of experimental procedures on the efficiency of learning and memory in the desert locust.

## 5. Conclusions

A robust method for assaying the effect of experimental procedures on the efficiency of learning and memory in the desert locust was developed. The role of short neuropeptide F in the regulation of these processes could not be demonstrated and remains the subject of further study.

## Supplementary Files

Supplementary File 1

## References

[1] Lee K.S., You K.H., Choo J.K., Han Y.M., Yu K. (2004). Drosophila short neuropeptide F regulates food intake and body size. J. Biol. Chem..

[2] Dillen S., Zels S., Verlinden H., Spit J., van Wielendaele P., Vanden Broeck J. (2013). Functional characterization of the short neuropeptide F receptor in the desert locust, schistocerca gregaria. PLOS ONE.

[3] Dillen S., Verdonck R., Zels S., van Wielendaele P., vanden Broeck J. (2014). Identification of the short neuropeptide F precursor in the desert locust: Evidence for an inhibitory role of sNPF in the control of feeding. Peptides.

[4] Marciniak P., Szymczak M., Rogalska L., Rosinski G. (2013). Developmental and myotropic effects of the Led-NPF-I peptide in tenebrionid beetles. Invertebr. Reprod. Dev..

[5] Cerstiaens A., Benfekih L., Zouiten H., Verhaert P., de Loofa A., Schoofs L. (1999). Led-NPF-1 stimulates ovarian development in locusts. Peptides.

[6] Johard H.A.D., Yoishii T., Dircksen H., Cusumano P., Rouyer F., Helfrich-Forster C., Nassel D.R. (2009). Peptidergic clock neurons in *Drosophila*: Ion transport peptide and short neuropeptide F in subsets of dorsal and ventral lateral neurons. J. Comp. Neurol..

[7] Chen W., Shi W., Li L., Zheng Z., Li T., Bai W., Zhao Z. (2013). Regulation of sleep by the short neuropeptide F (sNPF) in *Drosophila melanogaster*. Insect Biochem. Mol. Biol..

[8] Johard H.A.D., Enell L.E., Gustafsson E., Trifilieff P., Veenstra J.A., Nassel D.R. (2008). Intrinsic neurons of *Drosophila* mushroom bodies express short neuropeptide F: Relations to extrinsic neurons expressing different neurotransmitters. J. Comp. Neurol..

[9] Knapek S., Kahsai L., Winther A.M.E., Tanimoto H., Nassel D.R. (2013). Short neuropeptide F acts as a functional neuromodulator for olfactory memory in kenyon cells of *Drosophila* mushroom bodies. J. Neurosci..

[10] Davis R.L. (2011). Traces of *Drosophila* memory. Neuron.

[11] Stocker R.F., Lienhard M.C., Borst A., Fischbach K.F. (1990). Neuronal architecture of the antennal lobe in *Drosophila-melanogaster*. Cell Tissue Res..

[12] Nassel D.R., Enell L.E., Santos J.G., Wegener C., Johard H.A.D. (2008). A large population of diverse neurons in the *Drosophila* central nervous system expresses short neuropeptide F, suggesting multiple distributed peptide functions. BMC Neurosci..

[13] Krashes M.J., DasGupta S., Vreede A., White B., Armstrong J.D., Waddell S. (2009). A neural circuit mechanism integrating motivational state with memory expression in *Drosophila*. Cell.

[14] Root C.M., Ko K.I., Jafari A., Wang J.W. (2011). Presynaptic facilitation by neuropeptide signaling mediates odor-driven food search. Cell.

[15] Bernays E.A., Wrubel R.P. (1985). Learning by grasshoppers—Association of color light-intensity with food. Physiol. Entomol..

[16] Simoes P., Ott S.R., Niven J.E. (2011). Associative olfactory learning in the desert locust, Schistocerca gregaria. J. Exp. Biol..

[17] Raubenheimer D., Tucker D. (1997). Associative learning by locusts: Pairing of visual cues with consumption of protein and carbohydrate. Anim. Behav..

[18] Holliday J.L., Holliday N.J. (1995). Changes in learning-ability and mechanism during development of grasshopper nymphs, melanoplus-bivittatus. Physiol. Entomol..

[19] Simoes P.M.V., Ott S.R., Niven J.E. (2012). A long-latency aversive learning mechanism enables locusts to avoid odours associated with the consequences of ingesting toxic food. J. Exp. Biol..

[20] Levin E.D., Brucato F.H., Crapo J.D. (2000). Molecular overexpression of extracellular superoxide dismutase increases the dependency of learning and memory performance on motivational state. Behav. Genet..

[21] Helfrich-Förster C., Shafer O.T., Wulbeck C., Grieshaber E., Rieger D., Taghert P. (2007). Development and morphology of the clock-gene-expressing lateral neurons of *Drosophila melanogaster*. J. Comp. Neurol..

